# A Multifunctional Peptide Linker Stably Anchors to Silica Spicules and Enables MMP-Responsive Release of Diverse Bioactive Cargos

**DOI:** 10.3390/mi17010127

**Published:** 2026-01-19

**Authors:** So-Hyung Lee, Suk-Hyun Kwon, Byung-Ho Song, In-Gyeong Yeo, Hyun-Seok Park, A-Ri Kim, Lee-Seul Kim, Ji-Min Noh, Hee-Jung Choi, Da-Jeoung Lim, Young-Wook Jo

**Affiliations:** Department of Biotechnology, Yonsei University, Seoul 03722, Republic of Korea

**Keywords:** silica spicules, peptide–surface interactions, transdermal drug delivery, protease-cleavable linker, biomolecular interfaces, controlled release systems

## Abstract

Silica spicules provide a natural transdermal conduit but require a linker that binds strongly under physiological conditions and releases payloads selectively in response to biological cues. Existing silane chemistries or polydopamine coatings lack enzyme responsiveness and show limited control over release. We created a 180-member peptide library with the motif L–X1–X2–[Y–F–Y]–A–L–G–P–H–C and screened for silica binding. Biophysical assays (circular dichroism, ζ-potential, quartz crystal microbalance, atomic force microscopy) and molecular dynamics identified high-affinity binders. The lead, P176, was tested for matrix metalloprotease (MMP)-responsive cleavage. Conjugation and release of Vitamin C and Stigmasterol were analyzed by HPLC and Franz diffusion cells. P176 showed high silica affinity (~55 µg mg^−1^), robust biophysical signals (Δf −35 to −38 Hz; rupture force ~154 pN; ζ shift −22 to−11.5 mV), and favorable adsorption energy (−48.5 kcal mol^−1^, contact 4.5 nm^2^, 8.5 H-bonds). The MMP gate displayed efficient kinetics (Vmax 117.9 RFU·min^−1^, Km 5.0 µM) with >90% cleavage at 60 min, reduced to 26% by inhibitor. Conjugation yields reached 87% (Vitamin C) and 77% (Stigmasterol). Franz diffusion showed MMP-dependent release (24 h: Vitamin C 90–96%, Stigmasterol 80–85%) with minimal basal leakage. Released Vitamin C enhanced collagen I to ~250% in fibroblasts, while Stigmasterol attenuated LPS-induced macrophage morphology; keratinocytes retained normal marker expression. This study demonstrates that a single amphipathic, sequence-programmed peptide can couple strong silica anchoring with protease-responsive release and broad payload compatibility, establishing a versatile platform for spicule-based transdermal and regenerative delivery.

## 1. Introduction

Efforts to functionalize silica for biomedical use have largely relied on Tri-alkoxysilane chemistry (e.g., amino- and epoxy-silanes) to produce covalent monolayers that can subsequently tether ligands [1]. While effective on planar, rigorously dried substrates, these reactions require stringent hydrolysis/condensation control, organic solvents, and dehydration steps that are ill-suited to hydrated, rough microtopographies such as marine-derived spicules [2]. Silane films on irregular surfaces also suffer from variable grafting densities and hydrolytic instability, and they do not natively encode biochemical responsivity for on-demand release. More recent “universal” approaches—polydopamine or catechol coatings—improve adhesion breadth but form polymeric, redox-active layers with batch-dependent thickness and limited control over gate placement; release then requires an additional, orthogonal degradable element [3]. Related surface-immobilization fields have increasingly shifted from simple attachment chemistries toward linkers that control biomolecular orientation and/or enable stimulus-responsive activation, improving functional display at interfaces [4].

A parallel body of work has explored silica-binding peptides, discovered via phage display or inspired by diatom silaffin/R5 motifs [5]. These sequences are typically Lys/Arg-rich, bind silica by electrostatics and hydrogen bonding, and in some cases nucleate silica deposition [6]. However, their high cationic charge and conformational disorder make adsorption salt-sensitive and potentially nonspecific on biological interfaces; few reports quantify wash-resistant binding by QCM-D or single-molecule AFM, and fewer still integrate a protease-cleavable gate for responsive release. Where enzyme-labile motifs are used, they are often embedded in bulk hydrogels or brushes rather than interfacial linkers optimized for spicule microgeometries.

Stimuli-responsive release systems for the skin have primarily focused on MMP-sensitive prodrugs and hydrogels, leveraging elevated MMP activity in wound healing and inflammation [7]. Canonical motifs (e.g., GPQG or IWGQ variants) have been placed within polymer backbones or nanocarriers to achieve proteolysis-triggered unmasking or cargo liberation. Yet very few strategies unify a strong, noncovalent silica anchor that survives stringent washing on native spicules, a built-in MMP gate positioned for efficient proteolysis, and a modular conjugation handle compatible with chemically disparate payloads without altering the anchoring module [8].

This study addresses those gaps through a sequence-encoded, amphipathic linker (L–X1–X2–[Y–F–Y]–A–L–G–P–H–C) engineered to couple robust silica adsorption with MMP-responsive release. The design purposefully separates functions; an L–X–X hydrophobic nucleus and [Y–F–Y] electrostatic/aromatic block create an interfacial helix for strong, wash-resistant binding, and a G–P–H–C segment provides an MMP gate adjacent to a C-terminal Cys for thiol–maleimide conjugation. Unlike prior peptide binders, we combine library-level discovery (180 sequences) with mechanistic mapping (CD helicity, ζ-potential shifts, QCM-D Δf/ΔD, AFM rupture forces, and MD adsorption energetics) and then follow through to functional validation on spicules—not just planar silica—culminating in Franz-cell release and cell-level bioactivity.

We hypothesized that a single, sequence-programmed peptide can self-assemble into an amphipathic helix that binds silica/spicule surfaces with multipoint hydrophobic, electrostatic, and aromatic contacts sufficient to survive stringent washing; present an MMP-cleavable gate (G–P–H–C) in an orientation that supports rapid, inhibitor-sensitive proteolysis and thereby large on/off release ratios; and act as a modular platform whose C-terminal Cys enables high-yield conjugation to hydrophilic (Vitamin C) and hydrophobic (Stigmasterol) payloads, with released cargos retaining biological function in relevant skin-lineage cell assays. The ensuing experiments were designed to rigorously test each element of this hypothesis from binding physics to enzyme kinetics, release, and cellular efficacy on both planar silica and native spicules.

## 2. Materials and Methods

### 2.1. Peptide Synthesis and Quality Control

Peptides with the general sequence L–X1–X2–[Y–F–Y]–A–L–G–P–H–C (X1, X2 ∈ [F, W, V] [1]; Y ∈ [E, K] [9]) were synthesized by automated Fmoc solid-phase peptide synthesis (Liberty Blue™, CEM Corporation, Matthews, NC, USA) on Rink amide MBHA resin (0.6 mmol g^−1^, 0.1 mmol scale). Amino acids (4.0 eq) were activated with OxymaPure/DIC [10]. Difficult couplings, including hydrophobic double valine or aromatic clusters, were performed using HATU/DIPEA and double coupling cycles. Fmoc deprotection was carried out with 20% piperidine/DMF. Peptides were cleaved from resin using TFA:TIS:H_2_O:EDT (94:2.5:2.5:1, 2 h), precipitated with cold diethyl ether, washed, and lyophilized. Products were purified by reverse-phase HPLC (C18 column, 4.6 × 250 mm, 5 µm, gradient 5–60% acetonitrile/0.1% TFA over 20–30 min) and characterized by MALDI-TOF or LC–MS. Analytical HPLC confirmed ≥95% purity. For fluorescence assays, peptides were labeled at the N-terminus with FITC or biotin via an Ahx spacer, and labeling efficiency exceeded 95%.

### 2.2. Binding Assays on Silica and Spicules

For library screening, 1 mg of cleaned silica spicules was incubated with 1 µg of peptide in 200 µL HEPES buffer (10 mM HEPES, 150 mM NaCl, pH 7.4) at 25 °C for 30 min under rotation. Suspensions were centrifuged (3000× *g*, 5 min), and unbound peptide in the supernatant was quantified by fluorescence (Ex 485 nm, Em 520 nm). Adsorbed amounts were calculated relative to input. For surface spot assays, biotinylated peptides were spotted onto glass–silicate slides, dried, washed sequentially with 100 mM DTT, and detected using streptavidin–HRP chemiluminescence. For spicule imaging, FITC-labeled peptides (2 µM) were incubated with spicules, washed once or twice with PBS, and imaged by widefield or confocal fluorescence microscopy.

### 2.3. Biophysical Characterization

Quartz crystal microbalance with dissipation (QCM-D) was performed on a Q-Sense E4 instrument (Biolin Scientific AB, Gothenburg, Sweden)using SiO_2_-coated sensors (QSX303). Sensors were cleaned with piranha solution, equilibrated in buffer, and exposed to peptide solutions (50 µg mL^−1^, flow 0.1 mL min^−1^, 60 min, 25 °C). For silanol-blocked controls, sensors were pretreated with trimethylsilyl chloride.

Zeta potential measurements were performed on a Zetasizer Nano ZS (Malvern Panalytical Ltd., Malvern, UK) using silica suspensions (0.1 mg mL^−1^ in PBS, pH 7.4) incubated with peptides (50 µg mL^−1^, 30 min). Samples were centrifuged, resuspended, and analyzed using the Smoluchowski model.

Single-molecule force spectroscopy was performed with a Bruker Dimension Icon AFM (Bruker Corporation, Billerica, MA, USA) and MLCT cantilevers (Bruker Corporation, Billerica, MA, USA) (spring constant ~0.03 N m^−1^). Peptides were tethered to tips using NHS-PEG-maleimide (5 kDa) (Sigma-Aldrich, St. Louis, MO, USA) linkers through the terminal cysteine. Silica substrates were cleaned with piranha solution. Force curves were collected at 500–1000 nm s^−1^ with a 0.2–0.5 s contact time.

Circular dichroism spectroscopy was carried out on a Jasco J-815 spectrometer (JASCO Corporation, Tokyo, Japan) with 20 µM peptides in PBS (pH 7.4) using 0.1 cm cuvettes. Spectra were recorded between 190–260 nm at 50 nm min^−1^, 1 nm bandwidth, and averaged over three scans. CD spectra were analyzed primarily using the magnitude of the 222-nm minimum ([θ]222) as a comparative indicator of α-helical propensity across sequences. Detailed parameters for circular dichroism spectroscopy and molecular dynamics simulations are provided in Supplementary Table S1.

Molecular dynamics simulations were performed with GROMACS 2021.4 using the Amber99SB-ILDN (GROMACS, Stockholm, Sweden) force field. A β-cristobalite (001) slab (6 × 6 nm^2^, hydroxyl density 6.0 SiOH nm^−2^) was solvated with TIP3P water and 150 mM NaCl. After minimization and equilibration, production runs were carried out for 100 ns (2 fs timestep, 300 K, 1 bar). Adsorption energy, peptide–surface hydrogen bonds, and contact area were extracted from the final 20 ns.

### 2.4. Enzymatic Cleavage Assays

Recombinant human MMP-2 was activated with 1 mM APMA in assay buffer (50 mM Tris, 150 mM NaCl, 5 mM CaCl_2_, 0.05% Brij-35, pH 7.5). FRET substrates (0.5–20 µM) were incubated with 10 nM enzyme in black 96-well plates at 37 °C. Fluorescence (Ex 340 nm, Em 490 nm) was recorded, and initial velocities were fitted to the Michaelis–Menten equation. For time-course assays, substrates were tested at 10 µM with or without GM6001 inhibitor (25 µM). Cleavage reactions were analyzed by LC–MS (Agilent 6545 Q-TOF (Agilent Technologies, Santa Clara, CA, USA), BEH C18 2.1 × 100 mm, gradient 5–60% acetonitrile/0.1% formic acid over 20 min, 0.3 mL min^−1^).

### 2.5. Payload Conjugation and Release Assays

Peptides were reduced with 2 mM TCEP in PBS (pH 7.2, 30 min), desalted, and reacted with Mal-PEG_2_–Vitamin C or Mal-PEG_2_–Stigmasterol (1.5 eq) at room temperature for 2 h in the dark. Excess maleimide was quenched with 1 mM L-cysteine. Conjugates were purified and analyzed by HPLC (C18 column, 1 mL min^−1^, gradients 5–60% or 5–70% acetonitrile/0.1% formic acid). Yields were calculated from conjugate and parent peak areas. Assignments of conjugate peaks were based on the appearance of a new dominant RP-HPLC peak with concomitant depletion of the parent peptide peak.

Franz diffusion cells (diffusion area 0.64 cm^2^, PermeGear, Hellertown, PA, USA) were used with 5 mL receptor buffer (PBS, pH 7.4) at 32 °C under stirring (600 rpm). Donor chambers contained 500 µL conjugate solution (1 mg mL^−1^). For +MMP conditions, receptor buffer contained 50 nM MMP-2 or MMP-9. Aliquots were collected at 0–24 h and analyzed by HPLC or LC–MS.

### 2.6. Cell Culture and Functional Assays

HaCaT keratinocytes were cultured in DMEM with 10% FBS and 1% penicillin–streptomycin. Cells were treated with P176–Stigmasterol (30 µg mL^−1^) and immunostained for K14, FILAGGRIN, Phalloidin, and nuclei (DAPI).

Human dermal fibroblasts were cultured in DMEM with 10% FBS, seeded at 1 × 10^4^ cells/well in 96-well plates, and treated with P176–Vitamin C (50 µg mL^−1^) in the presence or absence of 50 nM MMP-2. After 48 h, collagen I levels were quantified by ELISA and visualized by immunofluorescence; viability was assessed by MTT assay. A 100 nmol ascorbate treatment served as positive control.

RAW264 macrophages were maintained in DMEM with 10% FBS and stimulated with LPS (100 ng mL^−1^) to induce inflammatory morphology. Cells were co-treated with P176–Stigmasterol (Sigma-Aldrich, St. Louis, MO, USA) and imaged by phase-contrast microscopy. Lipid-associated marker expression was measured by immunoassay, and LDH release was quantified using a CellTiter-Glo Luminescent cytotoxicity kit (Promega Corporation, Madison, WI, USA).

### 2.7. Statistical Analysis

All experiments were repeated at least three times. Data are presented as mean ± standard deviation. Comparisons were made using Student’s *t*-test or one-way ANOVA with Tukey’s post hoc test (GraphPad Prism version 9, GraphPad Software, San Diego, CA, USA). A value of *p* < 0.05 was considered statistically significant.

## 3. Results

### 3.1. Discovery of a High-Affinity Silica-Binding Peptide and Its Structural Signature

**Figure 1:**
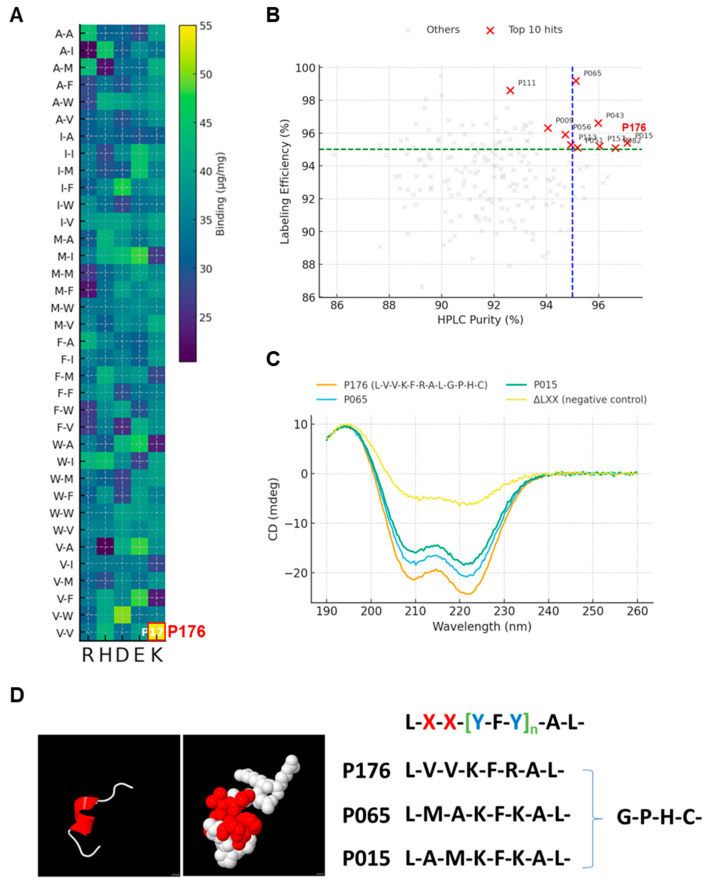
Identification and structural characterization of a silica-binding peptide linker. (**A**) Heat map of binding affinity (µg mg^−1^) from a 180-member peptide library with the consensus sequence L–X1–X2–[Y–F–Y]–A–L–G–P–H–C (X ∈ [F, W, V] [9]; Y ∈ [E, K] [9]). P176 (L-V-V-K-F-R-A-L-G-P-H-C) exhibited the highest binding (~55 µg mg^−1^). (**B**) Quality control plot of library members, showing HPLC purity versus labeling efficiency. Top-40% peptides clustered above 95% in both metrics, validating downstream analyses. (**C**) Circular dichroism spectra of top-40% peptides (*n* = 72) compared with negative controls (ΔLXX and [Y–A–Y]). Strong minima at 208 and 222 nm indicate α-helical structure in high-affinity sequences, whereas controls exhibited coil-like profiles. (**D**) Predicted structural models of representative peptides (P176, P065, P015) illustrating amphipathic helical organization. Hydrophobic residues (L, V, F, C) cluster on one face, while basic residues (K, R, H) align on the opposite face, rationalizing strong interfacial binding.

The 180-member library L–X1–X2–[Y–F–Y]–A–L–G–P–H–C displayed binding intensities ranging from ~22 to 55 µg mg^−1^ when normalized to spicule mass. The heat map shows a clear enrichment for V/I/W at X1/X2 and K/R at Y among the top-performing cells. The highest measured value was associated with P176 (V/V/K) at ~55 µg mg^−1^. This distribution motivated a top-40% subset (*n* = 72) for deeper analyses. Within this subset, sequences containing A at either X position were underrepresented, whereas double-branched aliphatic V/V was frequent, indicating a preference for compact hydrophobic packing at the interface (Figure 1A).

To exclude artifacts from impurities or labeling heterogeneity, we plotted HPLC purity versus labeling efficiency for the entire library. The top candidates clustered at 94–96% purity and 95–99% labeling, exceeding the thresholds (dashed lines at 95%) set a priori for downstream biophysical assays. P176 and several near-neighbors (e.g., P065) resided in the upper-right quadrant, providing confidence that subsequent differences stem from sequence rather than sample quality (Figure 1B).

CD spectra of the top-40% set exhibited a stereotyped double minimum at 208 nm and 222 nm with group averages [θ]208 = −16.2 ± 2.1 mdeg and [θ]222 = −20.5 ± 1.8 mdeg (*n* = 72), features diagnostic of α-helices. In contrast, the negative-control set (ΔLXX and [Y–A–Y]) produced shallow, broad minima (−4.5 ± 1.0 and −3.2 ± 0.9 mdeg), consistent with random-coil-rich ensembles. The magnitude of the 222-nm minimum, a proxy for helix content, aligned with binding rank order, suggesting that helical propensity supports surface attachment (Figure 1C).

Coarse-grained models of P176 and two representatives (P065, P015) folded to amphipathic helices in which L/V/F/C clustered on one face, with K/R/H oriented oppositely. This segregation rationalizes the heat-map bias toward V/V/K, and it provides a structural basis to expect strong hydrophobic contact and electrostatic complementarity to silanols and the hydrated silica layer (Figure 1D).

### 3.2. Robust Adsorption of Candidates to Silica and Spicule Surfaces

**Figure 2:**
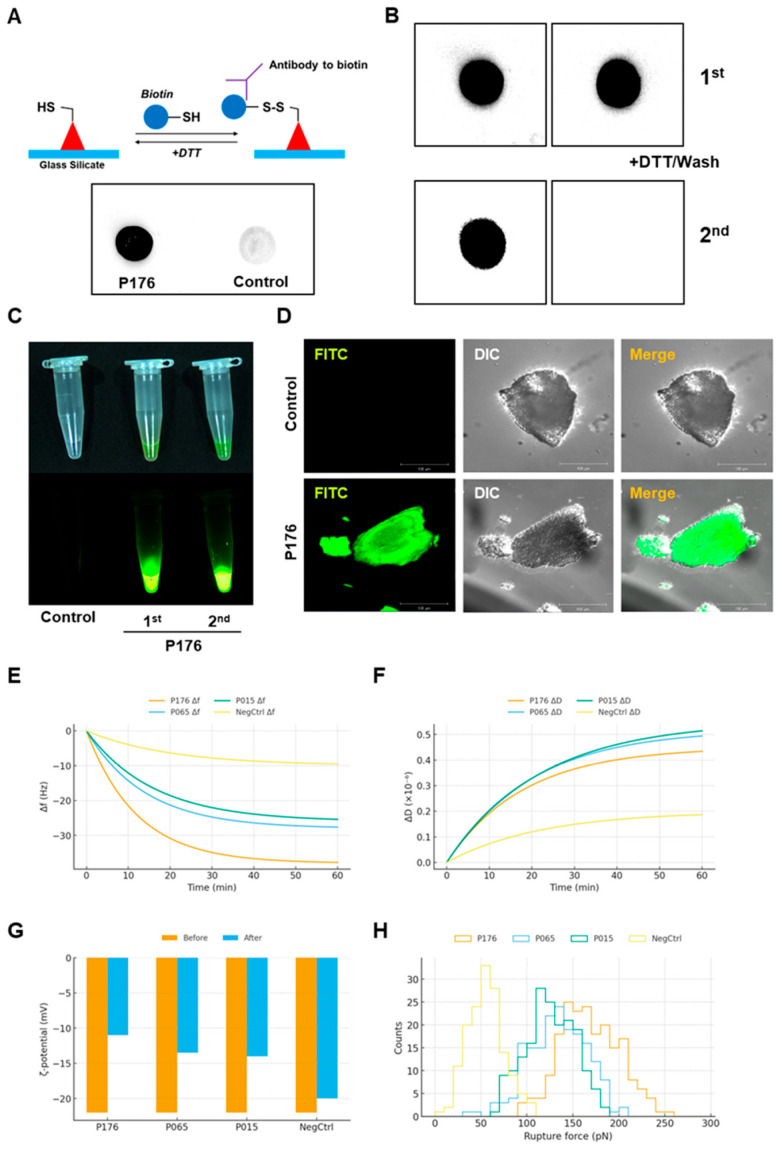
Verification of peptide adsorption to silica and spicule surfaces. (**A**) Biotin spot assay showing retention of P176 on glass–silicate surfaces after two sequential DTT washes, whereas the ΔLXX control was almost completely removed. Top panel: schematic of biotin labeling and detection; bottom panel: representative images of retained spots. (**B**) Wash stringency comparison between P176 and a low-affinity peptide (P015). Both peptides persisted after the first DTT wash, but only P176 remained visible after the second wash. (**C**) Fluorescence microscopy of FITC-labeled P176 bound to spicules after a single wash, showing strong residual signal. (**D**) Confocal microscopy at higher magnification confirming uniform distribution of FITC-P176 across spicule surfaces. (**E**,**F**) QCM-D traces on silica sensors. P176 produced a larger frequency shift (Δf ~−35 to −38 Hz) and lower dissipation than P065 or P015, consistent with denser, more rigid films. (**G**) ζ-Potential measurements showing silica baseline (−22 mV) shifted to −11.5 mV upon binding of top peptides (*n* = 72), but only to −19.8 mV for ΔLXX (*n* = 12). (**H**) AFM single-molecule force spectroscopy showing rupture forces of ~154 pN for top peptides versus ~58 pN for ΔLXX.

In a minimalist retention assay, spotted P176 remained strongly detectable after two sequential DTT washes, while ΔLXX largely disappeared. The schematic outlines biotin capture on thiols and anti-biotin detection; the contrasting black dot in the P176 panel against the faded control evidences wash-resistant adsorption. Because DTT disrupts disulfide-mediated bridging and weak hydrophobic interactions, P176’s survival indicates multipoint noncovalent anchoring beyond any incidental crosslinking (Figure 2A). We selected P015 from the binding map as a significantly lower-affinity comparator. After a 1st DTT wash, both P176 and P015 were present; after the 2nd wash, only P176 retained a strong spot, thereby recapitulating the ranking observed in Figure 1A and confirming that interfacial cohesion is sequence-encoded (Figure 2B).

FITC-tagged P176 incubated with spicules retained bright signal after 1× wash and remained detectable after 2× wash, mirroring the glass-silicate behavior. Confocal imaging at higher magnification showed uniform coverage across the rough spicule topography rather than punctate hotspots, indicating lateral mobility and self-averaging adsorption rather than isolated trapping (Figure 2C,D).

On pristine SiO_2_ sensors, P176 produced Δf ~ −35 to −38 Hz with ΔD ≈ 0.4–0.5 × 10^−6^, consistent with the deposition of a relatively rigid, compact film. P065 and P015 exhibited intermediate frequency shifts (approximately −20 to −25 Hz and −15 to −20 Hz, respectively), accompanied by smaller dissipation changes. On methylated silica, Δf values collapsed to ~−12 Hz (P176) and ~−4 Hz (ΔLXX), indicating a major dependence on silanol chemistry. The Voigt trends (large |Δf|, modest ΔD) agree with a tight interfacial assembly for high binders (Figure 2E,F).

The surface potential of silica suspensions shifted from −22.0 ± 0.7 mV to −11.5 ± 1.8 mV upon adsorption of the top-40% peptides (*n* = 72), while ΔLXX yielded only −19.8 ± 1.2 mV. The more positive potentials suggest K/R-driven charge compensation and reorganization of the electrical double layer, supporting the electrostatic component of the binding model (Figure 2G).

Individual unbinding events measured by AFM revealed a broad distribution centered at 153.9 ± 13.3 pN for the top-40% group (20 pulls/peptide) as compared with 57.8 ± 11.1 pN for ΔLXX. The absolute magnitudes are consistent with specific noncovalent clusters (multipoint hydrophobic/π/H-bond contacts) rather than purely electrostatic single-site interactions (Figure 2H).

### 3.3. Mechanistic Basis: Amphipathic Alignment and Cooperative Contacts

**Figure 3:**
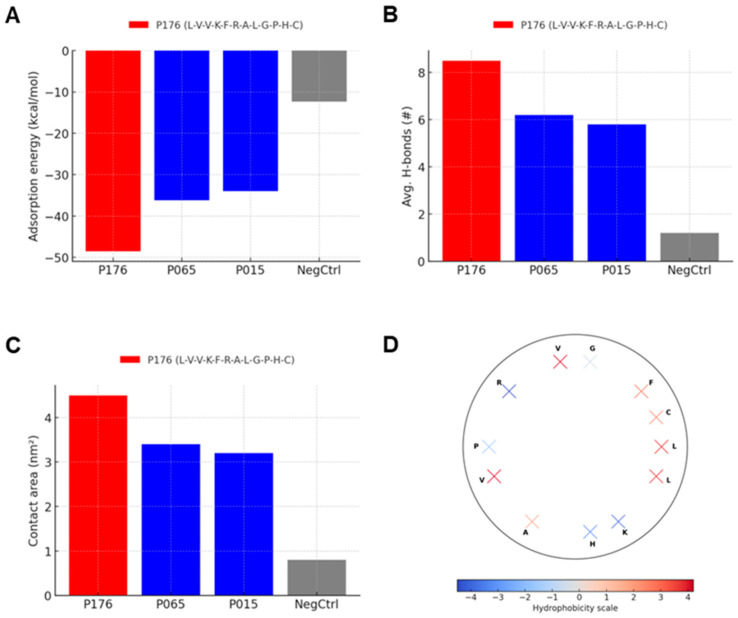
Mechanistic basis of silica binding. (**A**) Molecular dynamics simulations showing adsorption energies of P176 (−48.5 ± 3.2 kcal mol^−1^), P065/P015 (−36 to −38 kcal mol^−1^), and ΔLXX (−12.3 ± 2.5 kcal mol^−1^). (**B**) Number of peptide–surface hydrogen bonds, with P176 forming 8.5 ± 0.7 on average, compared to 5.8–6.2 for P065/P015 and ~1.2 for ΔLXX. (**C**) Interfacial contact area calculated from solvent-accessible surface area differences, showing P176 at 4.5 ± 0.3 nm^2^, larger than P065/P015 (3.2–3.4 nm^2^) and ΔLXX (0.8 ± 0.1 nm^2^). (**D**) Helical wheel projection of P176, illustrating amphipathic segregation of hydrophobic residues (L, V, F, C) on one face and basic residues (K, R, H) on the opposite face. This predicted model explains cooperative hydrophobic and electrostatic contacts underlying strong adsorption. Letters in the helical wheel (e.g., A, G, P) represent amino acid residues using the standard one-letter amino acid code.

In atomistic simulations on hydroxylated silica slabs, P176 exhibited an average adsorption energy of −48.5 ± 3.2 kcal mol^−1^, outcompeting P065/P015 (~−36 to −38 kcal mol^−1^) and ΔLXX (−12.3 ± 2.5 kcal mol^−1^). The larger magnitude for P176 is attributable to both van der Waals packing from the L–V–V face and electrostatic/π interactions mediated by K/R and F (Figure 3A). Analysis of hydrogen bonds formed between peptide side chains and surface silanols showed that P176 established an average of 8.5 ± 0.7 hydrogen bonds, markedly higher than the ~6 bonds observed for P065 and P015. ΔLXX produced only ~1.2 detectable hydrogen bonds, indicating negligible capacity for polar engagement with the silica network. The high number of hydrogen bonds for P176 reflects contributions from Lys, Arg, and His residues oriented toward the surface, and corresponds well with the ζ-potential shift observed experimentally (−22 to −11.5 mV). Calculation of solvent-accessible surface areas in contact with the silica slab revealed a contact area of 4.5 ± 0.3 nm^2^ for P176, substantially larger than 3.2–3.4 nm^2^ for P065 and P015 and 0.8 ± 0.1 nm^2^ for ΔLXX. This expanded footprint suggests that P176 flattens and maximizes interfacial interactions, consistent with the large QCM-D frequency shift (~−35 to −38 Hz) and high rupture forces (~154 pN) measured in experiments (Figure 3B,C).

A helical wheel representation of P176 illustrates a contiguous hydrophobic face composed of L, V, F, and C residues aligned on one side of the helix, opposite a basic face rich in K, R, and H. This amphipathic segregation predicts a peptide that can simultaneously bury hydrophobic residues against the surface while presenting polar/charged groups for hydrogen bonding and electrostatic interactions with silanols. Importantly, Figure 3D should be understood as a predicted model derived from structural projections, synthesizing the experimental and simulation data to provide a conceptual visualization of how sequence features drive the binding mechanism (Figure 3D).

### 3.4. Enzymatic Gate Performance: MMP Cleavage and Product Identity

**Figure 4:**
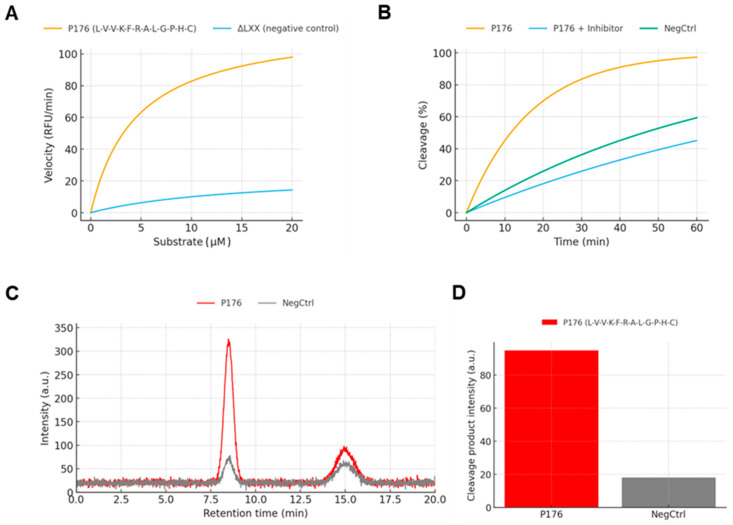
MMP-responsive cleavage of the peptide linker. (**A**) Michaelis–Menten kinetics of FRET-labeled substrates. P176 showed Vmax = 117.9 ± 12.3 RFU·min^−1^ and Km = 5.0 ± 1.1 µM, compared with Vmax = 26.3 ± 6.7 and Km = 15.5 ± 1.5 µM for ΔLXX. (**B**) Time-course cleavage at 10 µM substrate. P176 reached 92.7 ± 2.5% cleavage within 60 min in the presence of MMP, which was reduced to 26.7 ± 1.7% with GM6001 inhibitor. ΔLXX showed partial cleavage (52.9 ± 1.7%) and limited inhibitor sensitivity. (**C**) LC–MS chromatograms showing dominant product peaks at RT ~9–10 min for P176, with >3-fold higher intensity than ΔLXX, plus minor later-eluting fragments. (**D**) Quantification of integrated LC–MS peak areas confirming greater product yield for P176 relative to ΔLXX.

Michaelis–Menten analysis was performed using recombinant MMP-2 under defined assay conditions (50 mM Tris–HCl, 150 mM NaCl, 5 mM CaCl_2_, pH 7.5). Fluorescence traces from a FRET-labeled P176 substrate were recorded over a substrate range of 0.5–20 μM and fit globally to the Michaelis–Menten equation. P176 exhibited a Vmax of 117.9 ± 12.3 RFU·min^−1^ and a Km of 5.0 ± 1.1 μM, indicating both a high catalytic turnover rate and strong substrate affinity. By contrast, the ΔLXX control substrate displayed a Vmax of 26.3 ± 6.7 RFU·min^−1^ and a Km of 15.5 ± 1.5 μM, confirming that the absence of the hydrophobic nucleus markedly reduces enzymatic recognition efficiency. The nearly 5-fold higher Vmax and 3-fold lower Km of P176 establish that proper anchoring and helical ordering present the scissile G–P–H–C motif in an orientation that facilitates MMP access (Figure 4A).

To visualize cleavage dynamics, substrates were held at 10 μM and incubated with MMP-2 in the absence or presence of the broad-spectrum MMP inhibitor GM6001. For P176, cleavage increased rapidly and reached 92.7 ± 2.5% by 60 min under +MMP conditions, reflecting nearly complete processing within one hour. When GM6001 (25 μM) was pre-incubated, cleavage was strongly suppressed, plateauing at only 26.7 ± 1.7%. The ΔLXX control was cleaved less efficiently: under +MMP, the plateau was 52.9 ± 1.7%, and with inhibitor, cleavage was reduced to 13.3 ± 1.9%. These curves demonstrate two points: first, that P176 is cleaved more rapidly and to a higher extent than ΔLXX; second, that the process is specifically MMP-dependent and can be blocked pharmacologically, excluding spontaneous peptide degradation as a confounder (Figure 4B).

### 3.5. Payload Generality, High Conjugation Yields, MMP-Dependent Release, and Cytocompatibility

**Figure 5:**
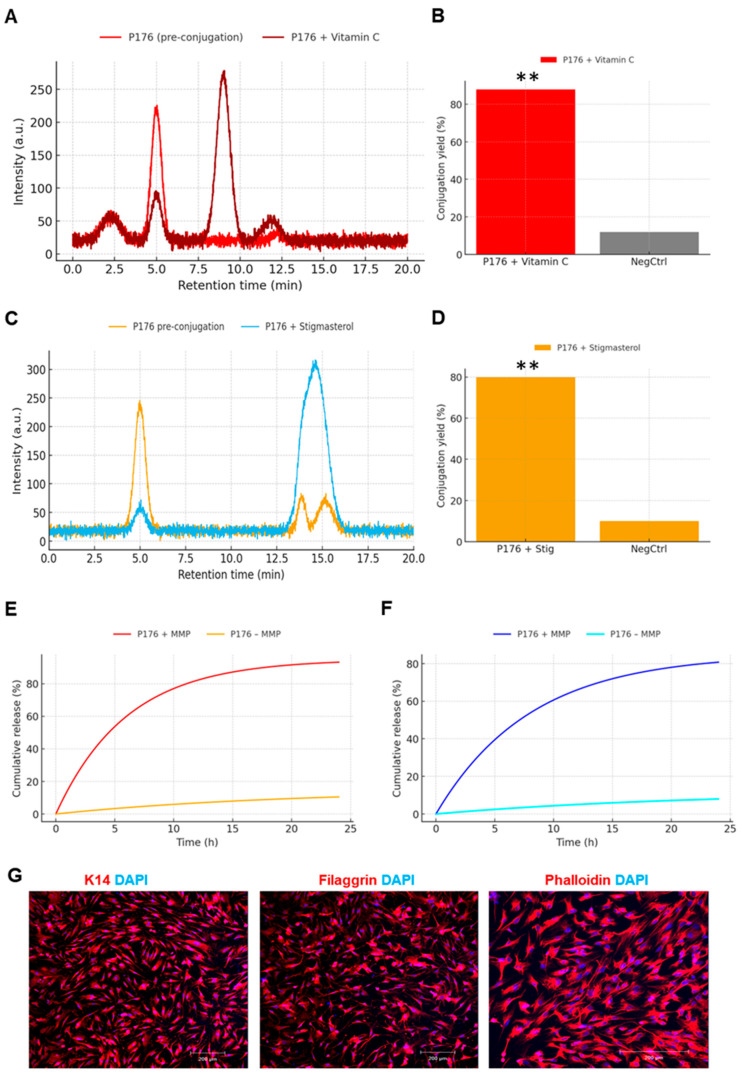
Conjugation of functional cargos and MMP-dependent release. (**A**) HPLC chromatograms of P176 before and after Vitamin C conjugation, showing appearance of a new peak at ~9 min and reduction of the parent peak at ~5 min. (**B**) Quantification of Vitamin C conjugation yields: P176 (86.9 ± 5.3%), other top peptides (~85–88%), and ΔLXX (~12%). *p* < 0.01 (**), as determined by Student’s *t*-test. (**C**) HPLC chromatograms of P176 before and after Stigmasterol conjugation, showing a new hydrophobic peak at ~14–15.5 min. (**D**) Quantification of Stigmasterol conjugation yields: P176 (77.3 ± 7.2%), other top peptides (~76–81%), and ΔLXX (~12%). *p* < 0.01 (**), as determined by Student’s *t*-test (**E**) Franz diffusion release profiles of Vitamin C conjugates. P176 released 90–96% over 24 h with MMP, versus 10–15% without MMP; ΔLXX released ~20% even with MMP. (**F**) Franz diffusion release of Stigmasterol conjugates. P176 released 80–85% under +MMP and 8–12% under −MMP, with slower kinetics than Vitamin C. (**G**) Confocal microscopy of keratinocytes treated with P176–Stigmasterol showing normal expression of K14, FILAGGRIN, and Phalloidin, indicating cytocompatibility.

Reverse-phase HPLC of P176 before and after conjugation to Vitamin C revealed a marked shift in retention profile. The parent peptide exhibited a major peak at ~5.0 min, accompanied by a minor early impurity peak. After conjugation, the parent peak was significantly reduced, and a new major peak appeared at ~9.0 min, corresponding to the more polar ascorbate conjugate. This retention time increase is consistent with the expected physicochemical change, as the ascorbyl moiety introduces additional hydrophilic functionality. The clear appearance of the new peak and concurrent depletion of the parent signal confirm successful conjugation (Figure 5A). Yields were calculated by integrating the areas under parent and conjugate peaks. For P176, the conjugation yield was 86.9 ± 5.3%, while four additional top peptides averaged 85–88%, demonstrating consistent high efficiency across lead candidates. By contrast, the ΔLXX control achieved only 12.7 ± 0.6%, confirming that disruption of the hydrophobic nucleus not only weakens surface binding but also interferes with productive Cys presentation in solution-phase coupling (Figure 5B).

Conjugation with hydrophobic Stigmasterol produced a distinct new peak at ~14–15.5 min, with broader width compared to the Vitamin C conjugate, reflecting its greater hydrophobicity and slower elution. The parent peptide peak was significantly diminished, again consistent with successful conjugation. The chromatographic profiles thus captured the physicochemical contrast between hydrophilic (Vitamin C) and hydrophobic (Stigmasterol) cargos, both efficiently attached to P176. Integration of HPLC peaks showed that P176 achieved 77.3 ± 7.2% yield for Stigmasterol. The other four top peptides produced similar values (76–81%), while the ΔLXX control remained low (11.8 ± 1.9%) (Figure 5C,D).

Release kinetics were assessed under +MMP and −MMP conditions. P176–Vitamin C conjugates released 90–96% of payload over 24 h in the presence of MMP, with an apparent half-time of ~2.3 h. In the absence of MMP, release plateaued at only 10–15%, demonstrating low basal leakage. The ΔLXX control released only ~20% even with MMP, indicating that weak binding and suboptimal gating severely impair payload liberation (Figure 5E).

Analogous experiments with Stigmasterol conjugates revealed 80–85% release under +MMP at 24 h, with a slower half-time of ~3.5 h, consistent with greater hydrophobic retention within the interfacial film. Under −MMP, release remained minimal (8–12%), and the ΔLXX control again failed to exceed ~20%. Despite slower kinetics relative to Vitamin C, the on/off ratio was maintained, demonstrating broad applicability of the MMP gate (Figure 5F).

Confocal microscopy of keratinocytes treated with P176–Stigmasterol showed normal morphology and marker expression. Immunostaining for K14, FILAGGRIN, and Phalloidin was indistinguishable from untreated controls, and nuclear morphology assessed by DAPI was intact. These results indicate that neither the peptide backbone nor the released Stigmasterol exerted detectable cytopathic effects under the tested conditions, supporting the cytocompatibility of the system (Figure 5G).

### 3.6. Bioactivity of Released Vitamin C and Stigmasterol

**Figure 6:**
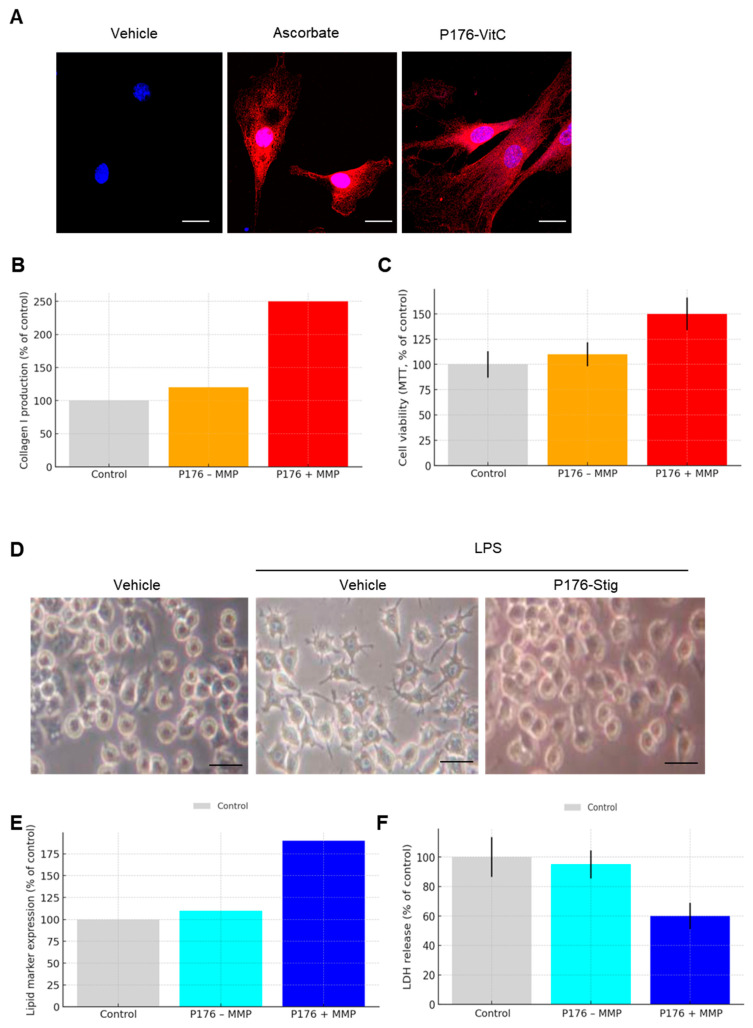
Bioactivity of released payloads. (**A**) Confocal images of fibroblasts treated with P176–Vitamin C in the presence of MMP, showing strong collagen I deposition comparable to free ascorbate (100 nmol). Scale bar = 10 µm. (**B**) Quantification of collagen I production by ELISA, reaching ~250% of vehicle under +MMP and ~110–135% under −MMP conditions. (**C**) Fibroblast viability assays showing no cytotoxicity under either condition. (**D**) Optical microscopy of RAW264 macrophages after LPS stimulation, showing dendritic spreading in controls but rounder morphology when co-treated with P176–Stigmasterol. (**E**) Biochemical assays confirming lipid marker upregulation and decreased LDH release in macrophages treated with P176–Stigmasterol under +MMP. Scale bar = 15 µm. (**F**) Summary of functional outcomes, demonstrating that both Vitamin C and Stigmasterol retain biological activity after conjugation and MMP-mediated release.

Fibroblasts treated with P176–Vitamin C in the presence of MMP displayed markedly increased collagen I deposition, visualized by confocal microscopy. The fluorescent signal intensity was comparable to that induced by 100 nmol free ascorbate (positive control), and much stronger than vehicle-treated or −MMP samples. The images showed dense, fibrillar collagen surrounding the cells, indicative of a functional pro-matrix effect (Figure 6A).

ELISA assays confirmed that collagen I levels reached ~250% of control in the +MMP group, compared with ~110–135% in the −MMP group. This twofold difference demonstrates that enzymatic release of Vitamin C restores its biological activity at levels comparable to direct supplementation, validating the functional integrity of the released cargo (Figure 6B). MTT assays showed no decrease in fibroblast viability after treatment with P176–Vitamin C conjugates under either +MMP or −MMP conditions. Cell survival remained at ~150% of untreated controls, excluding cytotoxicity as a cause for collagen induction (Figure 6C).

RAW264 macrophages exposed to LPS exhibited classic dendritic spreading and elongated processes consistent with inflammatory activation. In contrast, cells co-treated with P176–Stigmasterol displayed a rounder morphology with reduced dendrites, suggesting suppression of LPS-induced inflammatory responses. Optical microscopy clearly visualized this morphological difference (Figure 6D).

Quantitative assays of lipid-associated markers demonstrated upregulation under +MMP release, consistent with Stigmasterol activity, while LDH release was decreased relative to controls, indicating reduced cell damage. These findings support that enzymatically liberated Stigmasterol retained its functional anti-inflammatory effects (Figure 6E,F).

Together, the fibroblast and macrophage assays confirm that both hydrophilic (Vitamin C) and hydrophobic (Stigmasterol) cargos, once conjugated to P176 and released by MMP, are not only chemically intact but also biologically active in cell models directly relevant to dermal and inflammatory physiology.

## 4. Discussion

In this study, we designed, synthesized, and screened a 180-member peptide library to discover amphipathic motifs that can simultaneously provide strong adsorption to silica and controlled enzymatic release of conjugated payloads. From this library we identified P176 (L-V-V-K-F-R-A-L-G-P-H-C) as a top candidate based on binding affinity (≈55 µg mg^−1^), structural stability, and wash resistance. Biophysical assays including QCM-D, ζ-potential, AFM rupture force measurements, and circular dichroism confirmed that P176 forms a robust, amphipathic helical layer on silica and spicule surfaces, with anchoring driven by hydrophobic, electrostatic, and π-stacking interactions [6]. Molecular dynamics simulations supported this mechanism by showing favorable adsorption energies (−48.5 kcal mol^−1^), large contact areas (4.5 nm^2^), and multiple hydrogen bonds (8–9) with surface silanols [11]. To endow responsive release, the G-P-H-C motif was validated as an efficient MMP-cleavable gate, yielding high turnover (Vmax 117.9 RFU·min^−1^, Km 5 µM) and inhibitor-sensitive cleavage (>90% in 60 min, reduced to 26% with GM6001). Finally, conjugation of chemically distinct payloads—hydrophilic Vitamin C and hydrophobic Stigmasterol—proceeded in high yield (≈87% and 77%, respectively), and Franz diffusion assays confirmed MMP-dependent release with minimal basal leakage. Released cargos preserved bioactivity: Vitamin C induced collagen I upregulation (~250% of control) in fibroblasts, while Stigmasterol attenuated LPS-induced inflammatory morphology in macrophages without cytotoxicity in keratinocytes [12,13]. Together, these findings demonstrate a coherent platform where a single sequence-encoded peptide fulfills anchoring, gating, and conjugation functions for spicule-based delivery.

The results across multiple figures converge on the conclusion that the L-V-V hydrophobic nucleus is indispensable for adsorption. Heat maps (Figure 1A) revealed that peptides lacking branched hydrophobic residues showed weak binding [14], while those with double valines consistently ranked in the top 40%. CD spectra (Figure 1C) confirmed that these same peptides exhibited stronger α-helical character, and AFM rupture forces (Figure 2H) provided quantitative evidence that this helix mediates multipoint adhesion at ~150 pN per unbinding event [15]. Molecular simulations (Figure 3B,C) offered a mechanistic bridge, showing larger contact areas and increased hydrogen bonds for P176 compared with weaker binders (P015, P065). The ΔLXX variant consistently failed across assays—producing minimal frequency shifts in QCM-D, negligible ζ-potential changes, poor conjugation yield, and reduced MMP cleavage—underscoring that removal of the hydrophobic nucleus destabilizes both interfacial structure and enzymatic presentation.

Data from QCM-D and ζ-potential assays (Figure 2E–G) revealed that adsorption is not solely hydrophobic but requires cooperative amphipathic alignment. The shift in ζ-potential from −22 to −11.5 mV following peptide adsorption indicates that positively charged residues (Lys, Arg, His) directly interact with surface silanols, partially neutralizing the silica’s negative charge. AFM rupture forces supported this interpretation: events clustered at magnitudes typical of specific hydrogen-bonded and π-stacked contacts, rather than weak nonspecific electrostatics. Importantly, QCM-D on methylated silica produced only small frequency shifts (~−12 Hz), directly demonstrating that silanol groups mediate the high-affinity binding observed on hydroxylated surfaces [16]. Thus, P176’s amphipathic helix establishes a dual-face interaction: hydrophobic packing of L/V/F/C residues against surface water and siloxane patches, coupled with hydrogen bonding and charge interactions from K/R/H residues with silanol groups.

The G-P-H-C motif positioned downstream of the binding domain was hypothesized to serve as a protease-sensitive release site [17]. Kinetic assays (Figure 4A,B) validated this design, showing that P176 substrates were cleaved with higher efficiency (low Km, high Vmax) than ΔLXX, which presented the same scissile sequence but in a less ordered context [18]. Time-course assays demonstrated nearly complete cleavage (92.7%) within 60 min in the presence of MMP, while inhibitor treatment reduced Franz diffusion assays’ highlighted cleavage to 26.7%, confirming enzymatic specificity. LC–MS analysis (Figure 4C,D) corroborated these findings, showing dominant product peaks for P176 and only minor cleavage for ΔLXX. Together these results show that proper presentation of the cleavage motif by a well-anchored helix is crucial for efficient enzymatic access, linking surface anchoring directly to gate function. While this study establishes robust MMP-responsive behavior of P176, selectivity against non-target proteases was not systematically examined. Future work will address broader protease profiling; however, the modular design of the linker allows straightforward replacement or optimization of the cleavable motif to tailor enzyme specificity without altering silica anchoring.

HPLC analyses (Figure 5A,C) demonstrated successful conjugation of both hydrophilic Vitamin C and hydrophobic Stigmasterol, each producing distinct new chromatographic peaks at predicted retention times (~9 min and ~14–15.5 min, respectively). Quantification (Figure 5B,D) showed high conjugation yields for top peptides (85–88% Vit-C; 76–81% Stig) and low yields for ΔLXX (~10–13%). Franz diffusion assays (Figure 5E,F) highlighted the on/off performance of the MMP gate; under +MMP, >90% release of Vitamin C and ~80% release of Stigmasterol occurred within 24 h, while basal leakage under −MMP was ≤15%. The ΔLXX control, again, showed poor release (~20% under +MMP), underscoring the necessity of strong anchoring for effective gate function. These data establish that the peptide linker accommodates chemically diverse cargos without loss of release selectivity.

Together, these findings demonstrate that a single sequence-programmed peptide can integrate strong silica anchoring, enzymatic gating, and modular conjugation. Unlike silane chemistries or polydopamine coatings, this linker forms under aqueous conditions, maintains enzymatic responsiveness, and enables both hydrophilic and hydrophobic cargos [19]. Direct quantitative comparisons with APTES–glutaraldehyde or protein A-mediated immobilization were not performed, as these approaches operate through fundamentally different attachment chemistries and are optimized for different payload classes and performance metrics. Whereas silane and protein A strategies emphasize covalent grafting or protein orientation control, P176 was designed as a noncovalent, sequence-encoded linker that integrates strong adsorption on hydrated spicules with protease-gated release of small-molecule cargos. As such, rigorous head-to-head benchmarking would require a dedicated study with harmonized surface preparation and matched assay endpoints.

Potential applications include spicule-based transdermal delivery patches where elevated MMP activity at wound or inflamed sites controls release. Limitations remain: the current work used glass and isolated spicules, and in vivo environments with protein adsorption may alter interfacial binding. The gate was validated against MMP-2/-9, but selectivity versus other proteases must be confirmed. Nonetheless, the modular design permits substitution of alternative protease motifs for indication-specific responsiveness. Future work should extend to ex vivo human skin, evaluate pharmacodynamics in vivo, and explore scaling strategies for spicule coatings or microneedle integration. From a scalability standpoint, peptide synthesis cost and spicule variability represent practical considerations for future translation. Sequence minimization to identify the shortest functional anchoring–gating motif, together with substrate standardization using engineered silica microstructures, may improve manufacturability without altering the underlying mechanism demonstrated here.

## Data Availability

The original contributions presented in this study are included in the article/Supplementary Material. Further inquiries can be directed to the corresponding author.

## Supplementary Materials

The following supporting information can be downloaded at: https://www.mdpi.com/article/10.3390/mi17010127/s1, Table S1: Circular dichroism spectroscopy parameter table.

## References

[1] Sananes Israel S., Rebiscoul D., Odorico M., Flaud V., Ayral A. (2019). Surface Properties of Alkoxysilane Layers Grafted in Supercritical Carbon Dioxide. Langmuir.

[2] Grant S.R., Farach M.C., Decker G.L., Woodward H.D., Farach H.A., Lennarz W.J. (1985). Developmental expression of cell-surface (glyco)proteins involved in gastrulation and spicule formation in sea urchin embryos. Cold Spring Harb. Symp. Quant. Biol..

[3] Muhammad M., Ma R., Du A., Fan Y., Zhao X., Cao X. (2023). Preparation and Modification of Polydopamine Boron Nitride-Titanium Dioxide Nanohybrid Particles Incorporated into Zinc Phosphating Bath to Enhance Corrosion Performance of Zinc Phosphate-Silane Coated Q235 Steel. Materials.

[4] Yin T., Xu X., Huang Z., Rosa B.G., Gaboriau D.C.A., Merali N., Keshavarz M., Bin Muhammad Mustafa A.N., Song J., Alodan S. (2025). Control of Antibody Orientation on Graphene Using Porphyrin Linker Molecules for High-Performance Graphene-Based Immuno-Biosensors. J. Am. Chem. Soc..

[5] Thomassen A.B., Jansen T.L.C., Weidner T. (2024). The secondary structure of diatom silaffin peptide R5 determined by two-dimensional infrared spectroscopy. Phys. Chem. Chem. Phys..

[6] Naik R.R., Stringer S.J., Agarwal G., Jones S.E., Stone M.O. (2002). Biomimetic synthesis and patterning of silver nanoparticles. Nat. Mater..

[7] Corma A., Botella P., Rivero-Buceta E. (2022). Silica-Based Stimuli-Responsive Systems for Antitumor Drug Delivery and Controlled Release. Pharmaceutics.

[8] Kramer M.J., Leonard M.B., Ruan J., Lai D.E., Zavalij P.Y., Rodriguez E.E., Vedernikov A.N. (2025). Mesoporous Silica Nanoparticle Rigid Anchor Attached Pt Complex for Catalytic H/D Exchange of Aromatic Substrates. Inorg. Chem..

[9] Adler C., Monavari M., Abraham G.A., Boccaccini A.R., Ghorbani F. (2023). Mussel-inspired polydopamine decorated silane modified-electroconductive gelatin-PEDOT:PSS scaffolds for bone regeneration. RSC Adv..

[10] El-Faham A., Al Marhoon Z., Abdel-Megeed A., Albericio F. (2013). OxymaPure/DIC: An efficient reagent for the synthesis of a novel series of 4-[2-(2-acetylaminophenyl)-2-oxo-acetylamino] benzoyl amino acid ester derivatives. Molecules.

[11] Lechner C.C., Becker C.F. (2015). Silaffins in Silica Biomineralization and Biomimetic Silica Precipitation. Mar. Drugs.

[12] Nusgens B.V., Humbert P., Rougier A., Colige A.C., Haftek M., Lambert C.A., Richard A., Creidi P., Lapiere C.M. (2001). Topically applied vitamin C enhances the mRNA level of collagens I and III, their processing enzymes and tissue inhibitor of matrix metalloproteinase 1 in the human dermis. J. Investig. Dermatol..

[13] Gabay O., Sanchez C., Salvat C., Chevy F., Breton M., Nourissat G., Wolf C., Jacques C., Berenbaum F. (2010). Stigmasterol: A phytosterol with potential anti-osteoarthritic properties. Osteoarthr. Cartil..

[14] Tycko R. (2001). Biomolecular solid state NMR: Advances in structural methodology and applications to peptide and protein fibrils. Annu. Rev. Phys. Chem..

[15] Guan D., Hang Z.H., Marcet Z., Liu H., Kravchenko I.I., Chan C.T., Chan H.B., Tong P. (2015). Direct Measurement of Optical Force Induced by Near-Field Plasmonic Cavity Using Dynamic Mode AFM. Sci. Rep..

[16] Cheng D., Theivendran S., Tang J., Cai L., Zhang J., Song H., Yu C. (2022). Surface chemistry of spiky silica nanoparticles tailors polyethyleneimine binding and intracellular DNA delivery. J. Colloid Interface Sci..

[17] Nagase H., Visse R., Murphy G. (2006). Structure and function of matrix metalloproteinases and TIMPs. Cardiovasc. Res..

[18] Turk B.E., Huang L.L., Piro E.T., Cantley L.C. (2001). Determination of protease cleavage site motifs using mixture-based oriented peptide libraries. Nat. Biotechnol..

[19] Ryu J.H., Messersmith P.B., Lee H. (2018). Polydopamine Surface Chemistry: A Decade of Discovery. ACS Appl. Mater. Interfaces.

