# Supplementary material for: A Multifunctional Peptide Linker Stably Anchors to Silica Spicules and Enables MMP-Responsive Release of Diverse Bioactive Cargos
Source: Micromachines (Basel). 2026 Jan 19;17(1):127. doi: 10.3390/mi17010127 (PMC12844315; doi:10.3390/mi17010127)
Supplement: Supplementary file 1 [file micromachines-17-00127-s001.zip › micromachines-4055098-supplementary.pdf]

micromachines

MDPI

Table S1. Circular dichroism spectroscopy parameter table.

Category	Parameter	Value
Software	MD package	GROMACS 2021.4
Force field	Biomolecular force field	Amber99SB-ILDN
Surface model	Silica surface	β-cristobalite (001) slab
Surface dimensions	Slab size	6 × 6 nm^2
Surface chemistry	Hydroxyl density	6.0 SiOH nm^{-2}
Solvent	Water model	TIP3P
Ionic strength	Salt condition	150 mM NaCl
Workflow	Pre-production steps	Minimization and equilibration were performed prior to production runs
Production	Simulation length	100 ns
Integration	Timestep	2 fs
Thermodynamic conditions	Temperature	300 K
Thermodynamic conditions	Pressure	1 bar
Trajectory analysis	Analysis window	Final 20 ns of the trajectory
Outputs (reported)	Extracted observables	Adsorption energy; peptide–surface hydrogen bonds; contact area
